# Editorial: Exploring the interaction between health-promoting and health risk behaviours in health

**DOI:** 10.3389/fpubh.2024.1428819

**Published:** 2024-05-21

**Authors:** Feng Jiang, Huixuan Zhou, Yi-lang Tang

**Affiliations:** ^1^School of International and Public Affairs, Shanghai Jiao Tong University, Shanghai, China; ^2^Institute of Healthy Yangtze River Delta, Shanghai Jiao Tong University, Shanghai, China; ^3^Institute of Health Policy, Shanghai Jiao Tong University, Shanghai, China; ^4^Institute of Grand Health, Wenzhou Medical University, Wenzhou, China; ^5^Department of Physical Fitness and Health, School of Sport Science, Beijing Sport University, Beijing, China; ^6^Key Laboratory of Exercise and Physical Fitness, Ministry of Education, Beijing Sport University, Beijing, China; ^7^Department of Psychiatry and Behavioral Sciences, Emory University School of Medicine, Atlanta, GA, United States; ^8^Mental Health Service Line, Atlanta Veterans Affairs Medical Center, Decatur, GA, United States

**Keywords:** physical activity, health promotion, health-risk behaviors, lifestyle, interaction, health outcome

Health behaviors are pivotal determinants of individual wellbeing and are typically classified into health-promoting and health-risk behaviors (1). Understanding the complex interplay between these behaviors is crucial for designing effective health interventions and promoting overall health outcomes (2). In this Research Topic, the authors investigated the dynamic relationship between health promotion and health-risk behaviors in various health contexts.

One key aspect is the role of social and environmental factors in shaping health behaviors (3). Shi and Zhang underscored the influence of cultural beliefs on health attitudes and behaviors. Similarly, Wang et al. investigated the impact of social support on health-promoting behaviors among patients with type 2 diabetes mellitus. They highlighted the significance of interpersonal relationships in fostering positive health practices. Meanwhile, Huang et al. demonstrated that socioecological characteristics also affect health-promoting lifestyles.

Furthermore, health-promoting behaviors (Tao et al.), particularly physical exercise (Mu et al.), can mitigate negative emotions, yet strategies for improvement remain unclear (4). Zhang et al. designed an incentive program for promoting physical activity, revealing the multifaceted nature of behavior determinants. Yang et al. demonstrated that knowledge, attitudes, and practices were associated with health behaviors among middle school students. At the same time, Won and Shin indicated that health literacy was integral to health behaviors. These studies emphasize the importance of tailored approaches to address diverse population needs.

However, it is crucial to acknowledge the potential adverse consequences of certain behaviors on health outcomes (5). For instance, Qiu et al. revealed a positive association between television viewing and musculoskeletal disorders, which supports the known adverse effects of sedentary lifestyles. Similarly, findings from Jaswal et al., which linked alcohol consumption to smoking equivalents, and from Hou et al., which proved that unhealthy lifestyles linked with periodontitis, underscored the need to address multiple health risk behaviors comprehensively.

Moreover, interventions targeting specific populations, such as individuals with cardiovascular disease (Guo et al.), metabolic disorders (Jiang et al.), multiple sclerosis (Du et al.), or both cardiovascular and metabolic diseases (Li et al.), highlight the importance of personalized approaches in promoting health behaviors. These interventions encompass promoting physical activity, education, and socioeconomic factors influencing behavior change.

In conclusion, studies included in this Research Topic underscore the complex relationship between health-promoting and health-risk behaviors in shaping individuals' health trajectories. By shedding light on the intrinsic mechanisms and contextual elements that shape these behaviors, healthcare practitioners and policymakers can craft tailored interventions aimed at encouraging healthier lifestyles and alleviating the burden of preventable illnesses (6). Additionally, the content of this Research Topic could serve as a valuable material for textbooks on health behavior studies.

Future research should increasingly focus on promoting healthy behaviors and motivating individuals to take proactive actions toward their health, thereby effectively taking charge of their own health and wellbeing (7). This Research Topic requires a comprehensive exploration of multifaceted interventions spanning various domains such as education, healthcare, community engagement, and policy development. Utilizing innovative strategies grounded in behavioral science, technology, and social determinants of health, researchers can develop tailored approaches that resonate with diverse populations and address the underlying drivers of unhealthy behaviors. Furthermore, a multidisciplinary approach integrating insights from psychology, sociology, economics, and public health can offer holistic perspectives and novel solutions to encourage sustainable behavior change. Advancing knowledge in this domain is fundamentally important for nurturing a culture centered on health empowerment. Such a culture facilitates the empowerment of individuals to preserve their wellbeing. Consequently, this active engagement significantly contributes to the overall health of society.

## Author contributions

FJ: Conceptualization, Writing – original draft. HZ: Conceptualization, Writing – review & editing. Y-lT: Conceptualization, Writing – review & editing.
